# Comparative proteomic analysis of plasma from bipolar depression and depressive disorder: identification of proteins associated with immune regulatory

**DOI:** 10.1007/s13238-015-0218-5

**Published:** 2015-10-16

**Authors:** Jin Chen, ChengLong Huang, YiRen Song, HaiYang Shi, Dong Wu, YongTao Yang, ChengLong Rao, Li Liao, You Wu, JianYong Tang, Ke Cheng, Jian Zhou, Peng Xie

**Affiliations:** Department of Neurology, The First Affiliated Hospital of Chongqing Medical University, Chongqing, 400016 China; Chongqing Key Laboratory of Neurobiology, Chongqing, 400016 China; Institute of Neuroscience and the Collaborative Innovation Center for Brain Science, Chongqing Medical University, Chongqing, 400016 China; Department of Neurology, The Fifth People’s Hospital of Chongqing, Chongqing, 400016 China; Key Laboratory of Laboratory Medical Diagnostics of Education Ministry, Department of Laboratory Medicine, Chongqing Medical University, Chongqing, 400016 China; Department of Neurology, Yongchuan Hospital of Chongqing Medical University, Chongqing, 400016 China; Department of Cell Biology, Chongqing Key Laboratory of Neurobiology, Chongqing Medical University, Chongqing, 400016 China

**Dear Editor,**

Bipolar disorder (BD) is a debilitating psychiatric mood disorder affecting approximately 1%–3% of the population worldwide (Merikangas, 2007). Bipolar disorder is characterized by recurrent episodes of depression, hypomania, mania, or mixed states, and it has a poor outcome, with high rates of relapse, lingering residual symptoms, cognitive impairment, and functional impairment (Moreno et al., 2007) Although various etiopathological hypotheses concerning the disease have been reported, the pathophysiology underlying BD remains poorly understood (Gawryluk and Young, 2011).

According to the DSM-5 or ICD-10 criteria, the diagnosis of BD is still largely reliant on behavioral observations, and given the similarities in the clinical presentations between BD and unipolar major depressive disorder (MDD), especially bipolar II, approximately 40% of BD patients are initially misdiagnosed with MDD (Forte et al., 2015), and this may misguide the medication of BD patients. A better understanding of the pathophysiology underlying BD is thus essential to improve the diagnosis rate and pharmacotherapies for this disorder.

Proteomics, the quantitative analysis of protein expression in biosamples, is considered a powerful tool by which novel molecules and biomarkers of psychiatric disease can be identified (Roepstorff et al., 2012). Previous proteomic analyses of BD used mainly postmortem brain tissue, providing essential insight into BD (Behan et al., 2009); however, such analyses are inevitably affected by postmortem sample collection times. Antecedently, we performed comparative proteomics to distinguish protein expression differences between varying states of BD and healthy control (HC) subjects, and between MDD and HC groups (Song et al., 2015; Xu et al., 2012). To our knowledge, proteomic expression across BD and MDD has not been studied before; thus, there is an urgent need for this aspect to be investigated. The available evidence seems to indicate that brain imbalances can be reflected in the peripheral circulation (Herberth et al., 2010); using peripheral blood is therefore a practical means to achieving an improved understanding of the pathophysiological mechanisms underlying BD, with minimal collection risk and cost.

Here, a proteomic analysis based on two-dimensional electrophoresis (2-DE) coupled with matrix-assisted laser desorption/ionization-time-of-flight/time-of-flight tandem mass spectrometry (MALDI-TOF/TOF MS) was carried out on plasma samples from drug-naïve bipolar II and MDD patients, In order to overcome the limitations of a narrow linear range and large variations in 2-DE, 15 samples were mixed within each study group, and for each group, 2-DE was repeated in triplicate to limit run-to-run variations to about 20%, the criterion of 2-fold change was used as a cut off to ensure the reliability of apparent differences. PDQuest analysis revealed 2-fold differences for 25 distinct protein spots (*P* < 0.05). These 25 protein spots were successfully identified by MALDI-TOF MS/MS analysis (Fig. S1, Table S2). The expression levels of eight proteins were found to be significantly elevated and those of the other 17 proteins were significantly lower in bipolar II subjects compared with MDD subjects.

The DAVID Bioinformatics Resource v6.7 (http://david.abcc.ncifcrf.gov/home.js p) (Dennis et al., 2003) was used to obtain gene ontology (GO) terms and to carry out enrichment analysis to determine the most relevant GO and Kyoto encyclopedia of genes and genomes (KEGG) terms associated with the identified proteins. The cellular component (Fig. 1A) attributed to the differentially expressed proteins includes extracellular region, extracellular space, extracellular region part, high-density lipoprotein particle, plasma lipoprotein particle, and protein-lipid complex; and all the enriched biological processes (Fig. 1B) of the differentially expressed proteins were immune regulatory, including defense response, acute inflammatory response, response to wounding, and inflammatory response. All differently expressed proteins were mapped onto the KEGG database to determine the altered biological pathways associated with these plasma proteins. Notably, the complement and coagulation cascade pathways were enriched (*P* < 0.01) and five proteins were found to be involved in the pathway: C3, CFI, C4BPα,kininogen-1, and antithrombin. Among these, C3, CFI, and C4BPα were included in the pathway of complement cascades (*P* < 0.05).

**Figure 1:**
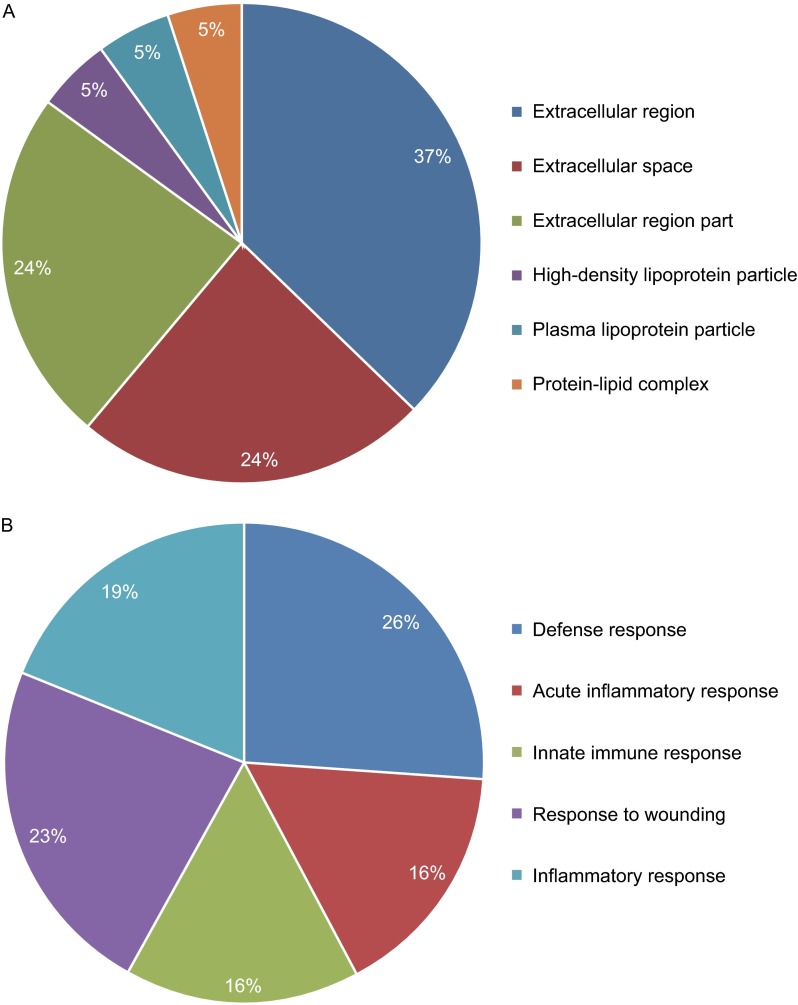
**Gene ontology**. The most significantly enriched (A) cellular component and (B) biological process according to DAVID Bioinformatics Resources based on the 25 differentially expressed proteins identified in bipolar II subjects relative to MDD subjects

Next, we choose the three significant differentially expressed complement proteins (C3, CFI, and C4BPα) for ELISA validation by using individual samples from the bipolar II (*n* = 20), MDD (*n* = 30), and HC groups (*n* = 30), plasma samples were diluted 1:10,000 for C3 analysis, 1:2000 for C4BPα analysis, and 1:1000 for CFI analysis. The quantitative results trends in expression patters were consistent with the MALDI -TOF/TOF MS findings, although the changes were smaller than those determined by 2-DE, the differences were statistically significant, and the expression levels of these three proteins being significantly dysregulated with the following profiles: C3, MDD > bipolar II > HC subjects; CFI and C4BPα, HC > MDD > bipolar II subjects (Fig. 2A–C).

**Figure 2:**
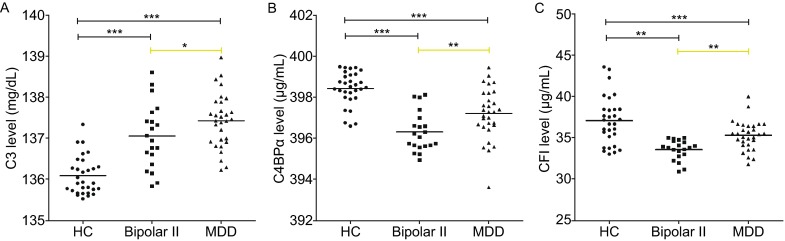
**Complement cascade-associated proteins analyzed by ELISA**. Significant differences between the bipolar II (*n* = 20), MDD (*n* = 30), and HC groups (*n* = 30) were observed. The expression levels of plasma C3 (A) in both the disease groups are significantly higher than the level in the HC group, and C3 expression was furthermore significantly upregulated in the MDD group compared with the bipolar II group. In contrast to C3, C4BPα (B) and CFI (C) expression levels were significantly downregulated in both disease groups compared to the HC group, the expression levels of these two proteins were significantly lower in bipolar II subjects compared to MDD subjects. *P < 0.05, **P < 0.001, and ***P = 0.000. Yellow lines highlight the differences between bipolar II and MDD subjects

The findings reported here provide evidence demonstrating that autoimmune dysregulation is involved in the pathophysiological mechanisms either bipolar II or MDD. Consistent with our findings, different lines of research have confirmed dysfunction in inflammation being closely related to the pathophysiology of BD and MDD (Leboyer et al., 2012). Our previous comparative proteomic analysis also showed immune dysregulation plays a role in the pathophysiology of BD and MDD (Song et al., 2015; Xu et al., 2012). C3 was considered as a significant feature of complement pathway activation because of its key role in all complement activation pathways (Jones et al., 2013). C4BP and CFI are important regulators of the complement system, which controls the activation of the complement cascades (Nilsson et al., 2011). The imbalance among them may bring autoimmune dysregulation.

Furthermore, as C3, C4BPα, and CFI display unique tiered expression profiles, the form and extent of the immune dysregulation underlying the two mental diseases may differ. The C3, CFI, and C4BPα proteins may represent a plasma-based diagnostic biomarker panel, which could aid BD diagnosis or be used to monitor BD progression. Current research appears to support the view that plasma levels of C3 are elevated in MDD patients, while changes in C3 in BD is closely associated with the state of disease (Santos Sória et al., 2012), and there is also mounting evidence of the C4BPα and CFI levels being disturbed in several psychiatric disorders, elevated peripheral C4BP levels have been reported in Alzheimer’s disease (Trouw et al., 2008), and the mean level of CFI activity in autistic subjects suggesting that high CFI activity may have an impact on the development of autism (Momeni et al., 2012). These findings seem to suggest that the autoimmune system may provide promising biomarkers for psychiatry. Further investigation into these proteins and the processes they are involved in is critical to gaining a better understanding of the underlying pathophysiology of BD.

## FOOTNOTES

We are grateful to the patients and control subjects that voluntarily participated in the study. Thanks to Professors Huaqing Meng and Delan Yang (Department of Psychiatry, First Affiliated Hospital, Chongqing Medical University) for their assistance in subject recruitment. This study was supported by the National Basic Research Program (973 Program) (Nos. 2009CB918300 and 2012CB910602).

Jin Chen, ChengLong Huang, YiRen Song, HaiYang Shi, Dong Wu, YongTao Yang, ChengLong Rao, Li Liao, You Wu, JianYong Tang, Ke Cheng, Jian Zhou, and Peng Xie declare that they have no conflict of interest.

All procedures followed were in accordance with the ethical standards of the responsible committee on human experimentation (institutional and national) and with the Helsinki Declaration of 1975, as revised in 2000 (5). Informed consent was obtained from all patients for being included in the study.

## Electronic supplementary material

Supplementary material 1 (PDF 314 kb)

Supplementary material 2 (PDF 46 kb)

Supplementary material 3 (PDF 90 kb)

Supplementary material 4 (PDF 79 kb)
