# Supplementary material for: Comparative proteomic analysis of plasma from bipolar depression and depressive disorder: identification of proteins associated with immune regulatory
Source: Protein Cell. 2015 Oct 16;6(12):908–11. doi: 10.1007/s13238-015-0218-5 (PMC4656209; doi:10.1007/s13238-015-0218-5)
Supplement: Supplementary file 1 — Supplementary material 1 (PDF 314 kb) [file 13238_2015_218_MOESM1_ESM.pdf]

MARERIALS AND METHODS

Subjects and samples

All subjects were recruited from the Psychiatric Center of the First Affiliated Hospital at Chongqing Medical University (Chongqing, China). Diagnoses were carried out using the Structured Clinical Interview from the DSM-IV-Axis I (SCID-I). All subjects were first-episode patients and were treatment-naïve. Bipolar II subjects were recruited if the patients complied with the standards of the Bech-Rafaelsen Mania Rating Scale (BRMS) ≥9 or with a 17-item version of the Hamilton Depression Rating Scale (HDRS) ≥17, while MDD patients were interviewed using the standards of the 17-item version of the Hamilton Depression Rating Scale (HDRS > 24). Potential subjects were excluded if they:

- were suffering from other mental illnesses or had a known history of mental disease such as schizophrenia and Parkinson's disease;
- had cerebral parenchymal disease or a history of brain trauma;
- hadan infectious or inflammatory disease;
- hada hepatic, renal, hematological, immunological, or thyroid disorder and/or severe complications from disease;
- had abnormal results in the laboratory examination of their blood, urine, or liver function;
- were female and were pregnant, lactating, or menstruating;
- were using any antidepressants or mood stabilizers

HC subjects were matched by age, gender, and BMI and were recruited from the Medical Examination Center of First Affiliated Hospital at Chongqing Medical University. In the period between 8:00 AM and 10:00 AM, fasting blood was collected using EDTA anticoagulant tubes, and samples were centrifuged at 14000 ×*g*

for 15 min to collect the plasma. The study sample set consisted of 35 bipolar II patients, 45 MDD patients, and 30 HC subjects. The detailed demographic data and clinic information of the recruited subjects are presented in supplemental table 1, There were no significant differences in the key demographic characteristics between the three groups (e.g., age, gender, or BMI), which consisted of BD patients, MDD patients, and healthy control (HC) subjects.

Immunodepletion of high abundance plasma proteins

Stored plasma samples were thawed and equal volumes of plasma from 15 bipolar II patients or 15 unipolar depression patients were pooled, respectively. According to the manufacturer's instructions, a multiple affinity removal column human-7 (4.6mm ID × 50mm; Agilent Technologies, Santa Clara, CA) was used to immunodeplete seven high abundance plasma proteins (fibrinogen albumin, IgG, haptoglobin, antitrypsin, IgA, and transferrin) from the pooled samples. A homemade C8 trapping column (4.6 mm ID × 10 mm, 5 m, 300 Å) was used to collect and desalt the flow-through fractions (low and medium abundance proteins). A SpeedVac (ThermoFisher Scientific RVT4104) was used to concentrate the desalted samples, after which 50 mM phosphate buffer (pH 8.5) containing 8 M urea was used to resuspend the samples. The Bradford method was used to determine the concentration of protein by using BSA as standard. The eluted fractions, flow-through fractions, and representative samples of crude plasma were separated by 12% SDS-PAGE and proteins were stained with silver as described previously in order to verify the efficiency of the immunodepletion (Yan et al., 2000).

Sample preparation

Fifteen samples per group (bipolar II and MDD groups) were pooled and 200 µl lysis buffer (7 M urea, 2 M thiourea, 4% CHAPS, 40 mM DTT, 0.2% Bio-Lyte 3/10

ampholytes) containing protease inhibitor (Sigma-Aldrich) was added to each sample. Samples were sonicated on ice (80 w, ultrasound 10 s, intermittent 15 s, a total of 10 times) and then centrifuged (30 min, 14000 × *g*). The resulting supernatants were collected and subjected to Bradford analysis to determine protein concentration.

2D Electrophoresis

For 2-DE, 100 μg protein was loaded onto analytical gels and another 400 μg onto preparative gels. Isoelectric focusing (IEF) was performed using the Ettan IPGphor Isoelectric Focusing System (GE Amersham) and pH 4-7 immobilized pH gradient (IPG) strips (13 cm, nonlinear; GE Healthcare). The IPG strips were rehydrated for 12 h in 250 μl rehydration buffer containing the protein samples and IEF was performed using the following conditions: 30V for 12 h, 500 V for 1 h, 1000 V for 1 h, and 8000 V for 8 h. Equilibration buffer (50 mM Tris-HCl [pH 8.8], 6 M urea, 2% SDS, 30% glycerol, and 1% DTT) was used to equilibrate the gel strips for 15 min. This step was repeated using the same buffer with 4% iodoacetamide instead of 1% DTT. The strips were then subjected to 2-DE after the proteins were transferred onto 12.5% SDS-polyacrylamide gels. Electrophoresis was performed using the Hofer SE 600 system (GE Amersham) at 15 mA per gel for 30 min, followed by 30 mA per gel, until the bromophenol blue reached the end of the gel. Three replicates were performed for each sample.

Gel staining and image analysis

Protein spots in the analytical gels were visualized by silver staining. The preparative gels were stained using modified silver staining methods compatible with subsequent mass spectrometric analysis (Yan et al., 2000).The analytical gels were scanned using an Epson 10000XL scanner (Epson Co., Ltd. Beijing, China) at an optical resolution of 300 dpi, and image analysis and spot detection were carried out

with PDQuest software version 8.0.1 (Bio-Rad Laboratories, Hercules, CA) using Gaussian spot modeling. Each paired spot was manually verified to ensure a high level of reproducibility between normalized spot volumes of gels produced in triplicate, and proteins with a 2-fold or greater overlap ratio threshold filtering were considered as differentially expressed proteins.

Two-dimensional gel excision, tryptic digestion, and desalting

Protein extracts were separated on preparative gels and proteins of interest were recovered from the gels for identification. Proteins (400 μg) from bipolar II and MDD groups were resolved on separate preparative polyacrylamide gels and were visualized by staining with modified silver staining methods compatible with subsequent mass spectrometric analysis. All differentially expressed spots were selected and cut from the four preparative gels after which 30 mM potassium ferricyanide/100mM sodium thiosulfate (1:1 v/v) was used to destain the excised protein spots for 20 min. The sports were then washed with Milli-Q water until they were destained. The spots were incubated in 0.2 M NH_4HCO_3 for 20 min and then lyophilized. Each spot was digested overnight in 12.5 ng/µl trypsin in 25 mM NH_4HCO_3. The peptides were extracted three times with 60% acetonitrile/0.1% trifluoroacetic acid and extracts were dried to completion and pooled using a vacuum centrifuge.

MALDI-TOF/TOF MS analysis

MS and MS/MS data for protein identification were obtained by using a MALDI-TOF-TOF instrument (5800 proteomics analyzer; Applied Biosystems). Instrument parameters were set using the 4000 Series Explorer software (Applied Biosystems) and MS spectra were recorded in reflector mode in a mass range from 800 to 4000 with a focus mass of 2000. CalMix5 standards were used to calibrate the instrument (ABI 4700 Calibration Mixture). For one main MS spectrum, 25

subspectra with 125 shots per subspectrum were accumulated using a random search pattern. For MS calibration, autolysis peaks of trypsin ([M+H]+842.5100 and 2,211.1046) were used as internal calibrators, and up to 10 of the most intense ion signals were selected as precursors for MS/MS acquisition, excluding the trypsin autolysis peaks and the matrix ion signals. In MS/MS positive ion mode, for one main MS spectrum 50 subspectra with 50 shots per subspectrum were accumulated using a random search pattern. The collision energy was 2 kV, the collision gas was air, and default calibration was set using the Glu1-Fibrino-peptide B ([M+H]+ 1,570.6696) spotted onto Cal 7 positions of the MALDI target. Combined peptide mass fingerprinting PMF and MS/MS queries was performed using the MASCOT search engine 2.2 (Matrix Science, Ltd.) embedded in the GPS-Explorer Software 3.6 (Applied Biosystems) on the NCBI database (download at Dec 31, 2014) with the following parameter settings: 100 ppm mass accuracy, trypsin cleavage (one missed cleavage allowed), carbamidomethylation set as fixed modification, oxidation of methionine was allowed as variable modification, MS/MS fragment tolerance was set to 0.4 Da. A GPS Explorer protein confidence index of ≥95% was used for further manual validation.

Bioinformatic analysis

The DAVID Bioinformatics Resource v6.7 (http://david.abcc.ncifcrf.gov/home.jsp) (Dennis et al., 2003) was used to obtain gene ontology (GO) terms and to carry out enrichment analysis to determine the most relevant GO and Kyoto encyclopedia of genes and genomes (KEGG) terms associated with the proteins of interest. All differentially expressed proteins were subjected to functional analysis using the gene ontology tool. The GO terms yielding $p < 0.05$ from a Fisher's exact test were considered significantly enriched. The KEGG pathways were deemed statistically

significant when they yielded a corrected $p < 0.05$, and these pathways were classified into hierarchical categories according to KEGG.

ELISA validation of proteins

In total, 80 individual plasma samples (20 bipolar II, 30 MDD, and 30 HC) were subjected to ELISA validation. Three differentially expressed proteins were analyzed using commercially available kits according to the manufacturers' instructions: complement component 3 (C3; Cloud-Clone Corp. Wuhan, China), Complement factor I (CFI; Cloud-Clone Corp.), and C4b-binding protein alpha (C4BPα; Cloud-Clone Corp.). No more than two freeze-thaw cycles were allowed per specimen. All samples were tested in triplicate and the mean concentrations were calculated.

Statistical analysis

Statistical analysis was performed using the Statistical Package of Social Science (SPSS) for Windows version 19.0. All data were expressed as mean ±SD. One-way analysis of analysis (one-way ANOVA) was applied to identify proteins with significant expression differences across the three groups. Differences with $p < 0.05$ were considered statistically significant.
