# Supplementary material for: Comparative proteomic analysis of plasma from bipolar depression and depressive disorder: identification of proteins associated with immune regulatory
Source: Protein Cell. 2015 Oct 16;6(12):908–11. doi: 10.1007/s13238-015-0218-5 (PMC4656209; doi:10.1007/s13238-015-0218-5)
Supplement: Supplementary file 2 — Supplementary material 2 (PDF 46 kb) [file 13238_2015_218_MOESM2_ESM.pdf]

Table 1. The basic demographic and clinical characteristics of subjects

Proteomic analysis

Variable	BD- II	MDD	BD-II/MDD
			p-value[a]
Sample size	15	15	—
Sex (M/F)	5/10	6/9	0.705
Age (year)[b]	32.06±7.20	34.12±11.27	0.557
BMI[b]	22.07±1.55	22.44±2.03	0.579
HAM-D scores[b]	22.41±2.81	24.02±5.20	0.300
BRMS scores[b]	10.23±2.41	—	—
Psychotherapeutic use			
Antipsychotics(Y/N)	0/15	0/15	1.000
Antidepressants(Y/N)	0/15	0/15	1.000
Mood stabilizers(Y/N)	0/15	0/15	1.000

ELISA Validation

Variable	BD- II	MDD	HC	BD- II / HC	MDD /HC	BD- II/MDD
				p-value[a]	*p*-value[a]	*p*-value[a]
Sample size	20	30	30	—	—	—
Sex (M/F)	9/11	12/18	13/17	0.907	0.793	0.726
Age (year)[b]	33.98±10.78	36.84±8.17	34.30±7.63	0.899	0.262	0.259
BMI[b]	21.72±4.56	21.73±4.57	22.99±9.10	0.507	0.995	0.463
HAM-D scores[b]	21.94±5.29	23.52±2.49	—	—	—	0.220
BRMS scores[b]	11.26±2.98	—	—	—	—	—
Psychotherapeutic use						
Antipsychotics(Y/N)	0/20	0/30	0/30	1.000	1.000	1.000
Antidepressants(Y/N)	0/20	0/30	0/30	1.000	1.000	1.000
Mood stabilizers(Y/N)	0/20	0/30	0/30	1.000	1.000	1.000

A Two-tailed student t-test for continuous variables (age, BMI, and HDRS Scores); Chi-square analyses for categorical variables (sex). Age, BMI, and HAM-D scores are presented as means ± SD's. Abbreviations: HC, healthy controls; BD- II, bipolar II disorder; MDD, unipolar major depressive disorder; M, male; F, female;

BMI, body mass index; HAM-D, Hamilton Depression Rating Scale; BRMS, Bech-Rafaelsen Mania Rating Scale; N, no; Y, yes.
