# Supplementary material for: Comparative proteomic analysis of plasma from bipolar depression and depressive disorder: identification of proteins associated with immune regulatory
Source: Protein Cell. 2015 Oct 16;6(12):908–11. doi: 10.1007/s13238-015-0218-5 (PMC4656209; doi:10.1007/s13238-015-0218-5)
Supplement: Supplementary file 3 — Supplementary material 3 (PDF 90 kb) [file 13238_2015_218_MOESM3_ESM.pdf]

Table 2. Differential proteins identified by MALDI-TOF/TOF MS

Spot No.	gi No.	Gene name	Protein name	Mascot score	Protein score C.I.%	MW [kDa]	pI	Biological function	Fold-change (BD II /MDD)
1	119598594	AHSG	alpha-2-HS-glycoprotein, isoform CRA_b	81	99.717	23147.6	4.81	Inflammation	7.38↑
2	340745278	HSF2	Heat shock factor protein 2 isoform c	73	97.854	27010.3	9.67	Inflammation	0.08↓
3	4502261	SERPINC1	Antithrombin-III precursor	250	100	53025	6.32	Blood coagulatio and inflammation	2.44↑
4	4504893	KNG1	Kininogen-1 isoform 2 precursor	134	100	48936.1	6.29	Blood coagulatio and inflammation	0.11↓
5	119598586	KNG1	Kininogen 1, isoform CRA_b	194	100	72953.5	6.34	Blood coagulatio and inflammation	0.47↓
6	19923106	PON1	Serum paraoxonase/arylesterase 1	82	99.696	39877.3	5.08	Lipid metabolism	0.1↓
7	262050546	KNG1	Kininogen-1 isoform 3 precursor	346	100	44763	6.07	Blood coagulatio and inflammation	2.5↑
8	139653	VTN	Vitronectin	80	99.591	55069.5	5.55	Immune	3.52↑
9	71773110	APOA4	Apolipoprotein A-IV precursor	97	99.992	45344.5	5.28	Lipid metabolism	0.33↓
10	41872583	ROCK2	Rho-associated protein kinase 2	77	98.844	161939.2	5.75	Apoptosis	0.43↓
11	119589476	C3	Complement component 3, isoform CRA_a	78	99.436	144417.1	8.24	Immune	0.47↓
12	119592981	A1BG	alpha-1-B glycoprotein	627	100	54808.8	5.58	Immune	3.59↑
13	4502133	APCS	Serum amyloid P-component precursor	333	100	25485.2	6.1	Inflammation	0.10↓
14	54036678	ACTG1	Actin, cytoplasmic 2	141	100	42107.9	5.31	Cell motility	0.27↓
15	74739412	POTEKP	Putative beta-actin-like protein 3	216	100	42331	5.91	Blood coagulation	3.2↑
16	116242809	SYNE2	Nesprin-2	70	95.181	801816.8	5.26	Maintain the subcellular spatial organization	0.45↓

17	119625323	FGG	Fibrinogen gamma chain, isoform CRA_a	91	99.969	38056.3	5.87	Blood coagulation	0.44↓
18	317373341	CFI	Complement factor I	199	100	68101.5	7.72	Immune	0.44↓
19	193806374	IGHM	Ig mu chain C region	90	99.962	49959.8	6.35	Immune	0.21↓
20	1708182	HPX	Hemopexin	586	100	52384.6	6.55	Transport	0.37↓
21	119604889	PGLYRP2	Peptidoglycan recognition protein 2, isoform CRA_b	270	100	68698.8	7.62	Immune	0.03↓
22	416733	C4BPA	C4b-binding protein alpha chain	218	100	69042.2	7.15	Immune	0.38↓
23	119589127	HPX	Hemopexin, isoform CRA_d	125	100	43771.5	6.24	Transport	7.74↑
24	530366454	C4BPA	PREDICTED: C4b-binding protein alpha chain isoform	210	100	69042.2	7.15	Immune	0.41↓
25	119619375	CXorf23	Chromosome X open reading frame 23, isoform CRA_b	72	97.236	77594.9	9.6	DNA replication	7.64↑

BD II, bipolar II disorder; MDD, unipolar major depressive disorde; MW, molecular weight; pI, isoelectric point.
