# Supplementary material for: Comparative proteomic analysis of plasma from bipolar depression and depressive disorder: identification of proteins associated with immune regulatory
Source: Protein Cell. 2015 Oct 16;6(12):908–11. doi: 10.1007/s13238-015-0218-5 (PMC4656209; doi:10.1007/s13238-015-0218-5)
Supplement: Supplementary file 4 — Supplementary material 4 (PDF 79 kb) [file 13238_2015_218_MOESM4_ESM.pdf]

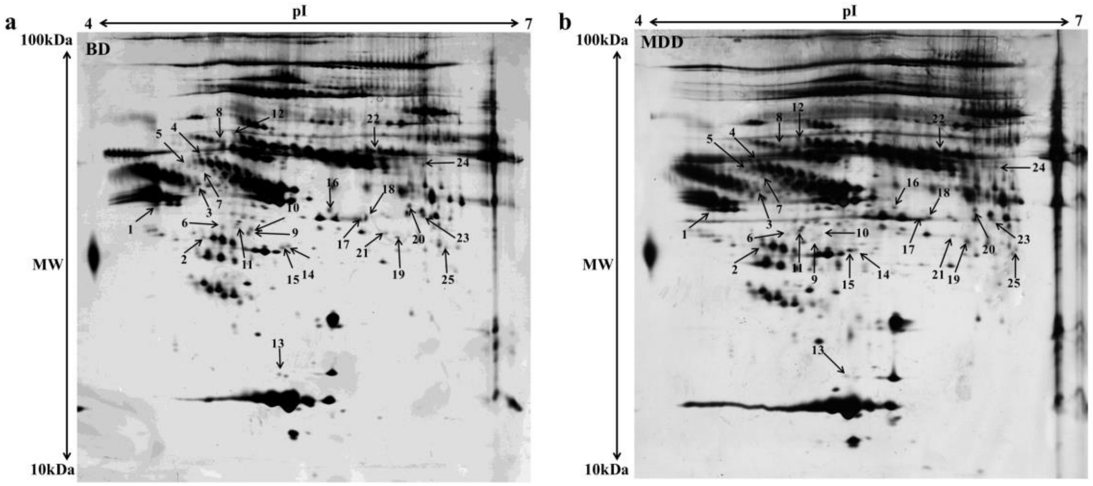

Supplementary figure 1. Representative silver-stained 2-DE gel images.

Differentially expressed proteins separated by 2-DE. Approximately 696 protein spots on gels with a 4-7 non-linear range were separated by silver staining. (a) 25 differential spots (numbered with arrows) were identified by MALDI-TOF/TOF MS in the bipolar II group. (b) 25 differential spots (numbered with arrows) were identified by MALDI-TOF/TOF MS in the MDD group. 2-DE, two-dimensional electrophoresis; IPG, immobilized pH gradient; SDS-PAGE, sodium dodecyl sulfate polyacrylamide gel electrophoresis; MALDI-TOF/TOFMS, matrix-assisted laser desorption/ionization time-of-flight/time-of-flight mass spectrometry.
